# An Improved Genome-Wide Polygenic Score Model for Predicting the Risk of Type 2 Diabetes

**DOI:** 10.3389/fgene.2021.632385

**Published:** 2021-02-11

**Authors:** Wei Liu, Zhenhuang Zhuang, Wenxiu Wang, Tao Huang, Zhonghua Liu

**Affiliations:** ^1^Department of Statistics and Actuarial Science, The University of Hong Kong, Hong Kong, China; ^2^Department of Epidemiology and Biostatistics, School of Public Health, Peking University, Beijing, China; ^3^Center for Intelligent Public Health, Institute for Artificial Intelligence, Peking University, Beijing, China; ^4^Key Laboratory of Molecular Cardiovascular Diseases, Ministry of Education, Peking University, Beijing, China

**Keywords:** type 2 diabetes, UK Biobank, screening, prediction model, polygenic risk score

## Abstract

Polygenic risk score (PRS) has been shown to be predictive of disease risk such as type 2 diabetes (T2D). However, the existing studies on genetic prediction for T2D only had limited predictive power. To further improve the predictive capability of the PRS model in identifying individuals at high T2D risk, we proposed a new three-step filtering procedure, which aimed to include truly predictive single-nucleotide polymorphisms (SNPs) and avoid unpredictive ones into PRS model. First, we filtered SNPs according to the marginal association *p*-values (*p*≤ 5× 10^−2^) from large-scale genome-wide association studies. Second, we set linkage disequilibrium (LD) pruning thresholds (*r*^2^) as 0.2, 0.4, 0.6, and 0.8. Third, we set *p*-value thresholds as 5× 10^−2^, 5× 10^−4^, 5× 10^−6^, and 5× 10^−8^. Then, we constructed and tested multiple candidate PRS models obtained by the PRSice-2 software among 182,422 individuals in the UK Biobank (UKB) testing dataset. We validated the predictive capability of the optimal PRS model that was chosen from the testing process in identifying individuals at high T2D risk based on the UKB validation dataset (*n* = 274,029). The prediction accuracy of the PRS model evaluated by the adjusted area under the receiver operating characteristics curve (AUC) showed that our PRS model had good prediction performance [AUC = 0.795, 95% confidence interval (CI): (0.790, 0.800)]. Specifically, our PRS model identified 30, 12, and 7% of the population at greater than five-, six-, and seven-fold risk for T2D, respectively. After adjusting for sex, age, physical measurements, and clinical factors, the AUC increased to 0.901 [95% CI: (0.897, 0.904)]. Therefore, our PRS model could be useful for population-level preventive T2D screening.

## Introduction

Type 2 diabetes (T2D) is a global public health problem. Identifying individuals at high risk for T2D for early targeted detection, prevention, and intervention is of great public health significance. Besides the well-known behavioral and environmental factors, T2D has a strong genetic component (Zimmet et al., 2014). Genome-wide association studies (GWASs) have successfully identified many common genetic variants that confer T2D susceptibility (Burton et al., 2007; Scott et al., 2007; Palmer et al., 2012; Visscher et al., 2017; Pärna et al., 2020). However, all of these common genetic variants discovered by GWAS can only be able to account for a small proportion of the total heritability (McCarthy, 2010; Herder and Roden, 2011; Prasad and Groop, 2015) and thus lead to low predictive power. Polygenic risk score (PRS) that aggregates the information of many common single-nucleotide polymorphisms (SNPs) weighted by the effect size obtained from large-scale discovery GWAS has been used to predict T2D risk. PRS is expected to have better predictive power and the potential to improve the performance in T2D risk assessment (Wray et al., 2013; Khera et al., 2019).

The most commonly used method for constructing PRS is called clumping and thresholding (C + T) [or pruning and thresholding (P + T)] method, which applies two filtering steps. To retain SNPs that weakly correlated with each other, it first forms clumps around SNPs by using linkage disequilibrium (LD)-driven clumping procedure (Privé et al., 2019). Each clumping contains all SNPs within 250 kb of the index SNPs, and the degree of LD is determined by a provided pairwise correlation (*r*^2^). Then, it removes SNPs with *p*-values obtained from a disease-related GWAS larger than a given threshold. C+T is regarded as the most intuitive and easiest method to generate PRS. There are two common software programs (i.e., PLINK and PRSice) that can be used to implement C + T method. Recently, Choi et al. developed a new software PRSice-2 from https://www.prsice.info (Choi and O’Reilly, 2019), which is demonstrated to be more computationally efficient and scalable than alternative PRS software while maintaining comparable predictive power.

Several researchers have tried to construct PRS models based on the C + T method for predicting T2D risk by PLINK or PRSice software. The earliest PRS model assessed the combined risk of only three variants that had been published to predispose to T2D in 6,078 individuals. The area under the receiver operating characteristics curve (AUC) of their PRS model was 0.571 (Weedon et al., 2006). Thereafter, other researchers have attempted various strategies to improve the predictive ability of the PRS model, including increasing the number of SNPs, adjusting for sex and age, some physical measurements [e.g., body mass index (BMI), diastolic blood pressure (DBP), and systolic blood pressure (SBP)] (Lango et al., 2008) and clinical factors [e.g., triglyceride level (TL), glucose level (GL), and cholesterol level (CL)] (Lyssenko et al., 2008; Meigs et al., 2008; Vassy et al., 2014). The AUC of those improved PRS models increased to some extent (range from 0.600 to 0.800). However, there are still several limitations. First, their sample sizes are not large (range from 2,776 to 39,117). Second, they only take a small number of SNPs (range from 3 to 1,000) that passed the “GWAS significant variant” derivation strategy (*p*≤ 1× 10^−8^ and *r*^2^ < 0.2) into account, which is too strict and might miss predictive SNPs. Amit et al. (Khera et al., 2018) constructed the PRS model across the whole genome and finally included a total of 409,258 individuals with 6,917,436 SNPs from the UK Biobank (UKB) project. The AUC was 0.730 after adjusting for age, sex, and the first four principal components for ancestry. This strategy has a slight improvement in prediction accuracy; however, the computational burden is relatively large.

To further explore the prediction capability of the PRS model in identifying high-risk individuals for T2D, we proposed a new strategy to construct PRS model by the following three-step filtering procedure to consider a statistical compromise between signal and noise. First, rather than including SNPs across the whole genome, we selected a subset of SNPs by a lenient significance threshold (*p*≤ 5× 10^−2^) from a huge number of SNPs included in large-scale GWASs. Second, we set *r*^2^ equal to 0.2, 0.4, 0.6, and 0.8 as candidate LD pruning thresholds according to Khera et al. (2018). Third, we set *p*-value thresholds as 5× 10^−2^, 5× 10^−4^, 5× 10^−6^, and 5× 10^−8^. After applying the above thresholds to the GWAS summary data, a total of 16 candidate PRS models were then generated based on the PRSice-2 software in the target samples. We conducted testing using the UKB testing dataset (*n* = 182,422) to avoid the model overfitting issue. Finally, we chose the best predictive PRS model among a set of candidate PRS models and evaluated it in the UKB validation dataset (*n* = 262,751). We also considered non-genetic risk factors, including sex, age, physical measurements, and clinical factors to further increase prediction accuracy. Real data analysis showed that our PRS model outperforms previous prediction models for T2D.

## Materials and Methods

### Study Design and Population

Our study was conducted based on the UKB project^1^, one of the largest prospective cohort studies (Conroy et al., 2019). Nearly half a million participants aged 40–69 years were enrolled from the United Kingdom at the time of their baseline assessment visited from 2006 to 2010 (Sudlow et al., 2015). A wide kind of physical measures (e.g., height, weight, blood pressure, and spirometry) and biological samples (e.g., blood, urine, and saliva) were collected. It then converted the limited information contained in the biological samples into widely shared cohort-wide genotyping (Bycroft et al., 2018) and whole-exome sequencing data (Khera et al., 2019). More details about the study design, method, and participants of the UKB project have been provided elsewhere (Sudlow et al., 2015).

A total of 487,409 individuals with available genotyping array and altogether 625,394 variants were originally collected from UKB. We conducted strict quality control (QC) steps described by Marees et al. (2018) based on PLINK 2.0 from https://www.cog-genomics.org/plink2. Specifically, we first filtered out SNPs and individuals with very high levels of missingness. Based on a relaxed threshold of 0.2 (>20%), we removed 89,752 variants and 30,855 subjects. There were also 262,751 SNPs removed with minor allele frequency <0.03 and 1,204 SNPs removed with a *p*-value of Hardy–Weinberg equilibrium Fisher’s exact test < 1×10^−6^. Finally, 456,451 individuals and 271,687 variants passed QC and were considered in the following analysis.

**FIGURE 1:**
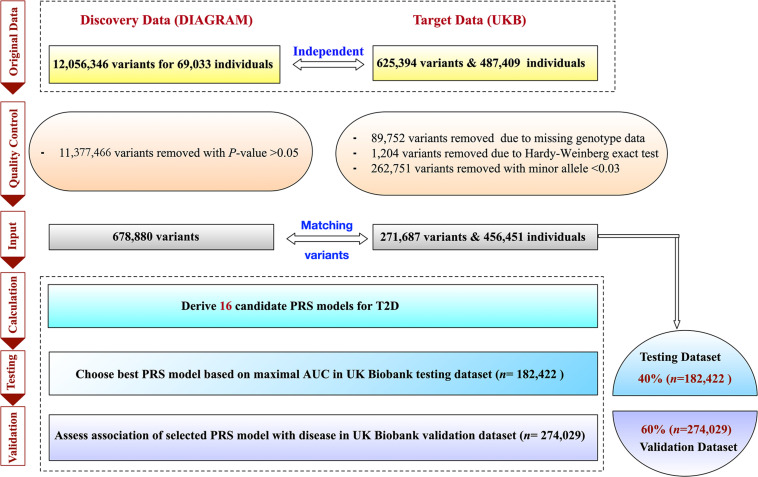
Flowchart for the polygenic risk score (PRS) model for type 2 diabetes.

The ascertainment of T2D was based on a composite of self-report, the International Classification of Diseases, Ninth Revision (ICD-9) codes of 25000 and 25010, and the International Classification of Diseases, Tenth Revision (ICD-10) code of E11. The individual-level data of T2D-related risk factors, including sex, age, physical measures [e.g., BMI, waist circumference (WC), DBP, and SBP] and clinical factors [e.g., GL, CL, TL, high-density lipoprotein (HDL), low-density lipoprotein (LDL)] were also collected from the UKB project. We further imputed the inevitably missing values of these factors by their means. To analyze individuals with a relatively homogeneous ancestry, the population was constructed centrally based on a combination of self-reported ancestry and genetically confirmed ancestry using the first 10 principal components (i.e., PC_1_,…,PC_10_). To construct, test, and further validate the robustness of the polygenic predictor of T2D, we randomly divided the overall data into two parts, i.e., the testing and validation dataset. We assigned 40% of all individuals as the UKB testing dataset (*n* = 182,422) and the remaining 60% as the UKB validation dataset (*n* = 274,029). Other ratios were also tried to divide the testing and validation datasets, i.e., 30–70%, 50–50%, 60–40%, and 70–30%. Individuals in the UKB validation dataset were distinct from those in the UKB testing dataset. The detail of the study design is described in Figure 1.

### Genome-Wide Polygenic Score Construction, Testing, and Validation

The PRS model provides a quantitative metric of an individual’s inherited risk based on the cumulative impact of many SNPs. Generally, the PRS model can be unweighted or weighted. Suppose that we have *n* subjects and *K* SNPs that passed the first-step filtering procedure. The unweighted PRS model is defined as,

$${\text{PRS}}_{u}=G_{1}+\ldots .,G_{K},$$

where *G*_*k*_(*k* = 1,….,*K*) denotes the number of risk alleles for each genetic variant coded as 0, 1, or 2 under the additive genetic model. For the weighted PRS model, weights are generally assigned to each genetic variant according to the strength of association with a given disease. The weighted PRS model can be written as,

$${\text{PRS}}_{w}={\hat{\beta }}_{1}\,G_{1}+\text{…},{\hat{\beta }}_{K}\,G_{K},$$

where ${\hat{\beta }}_{k}\left(k=1,\text{…},K\right)$ is the estimate of marginal genetic effect in the external large-scale GWAS. Both unweighted or weighted PRS models can be implemented by the PRSice-2 software (Choi and O’Reilly, 2019).

For PRS model construction, we used summary statistics from a T2D GWAS conducted among 60,786 participants with 12,056,346 SNPs of European ancestry^2^ (Morris et al., 2012). Note that the UKB samples did not overlap with the samples from discovery GWAS. We first selected SNPs according to their association *p*-values (*p*≤ 5× 10^−2^) obtained from the above GWAS, and 50,224 SNPs remained. We then considered multiple *r*^2^ thresholds (0.2, 0.4, 0.6, and 0.8) according to Khera et al. (2018) and *p*-value thresholds (5× 10^−2^,5× 10^−4^,5× 10^−6^, and 5× 10^−8^) to conduct the second and third filtering procedures also on the DIAGRAM summary dataset. A total of 16 candidate PRS models were created for T2D based on the UKB testing dataset with 182,422 participants.

The PRS model with the best discriminative accuracy was determined based on the maximal AUC in the following logistic regression model adjusting for sex, age, and the first 10 principal components of ancestry. We use *X*_1_,*X*_2_ and **PC** = (PC_1_,…,PC_10_)^*T*^ to represent the value of sex, age, and the first 10 principal components of ancestry, respectively, where *T* denotes the transpose of a vector or matrix. Let *Y* be the T2D status with 0 and 1 representing control and case. The predictive model for T2D can be represented as,

$$\text{Logit}\left[P\left(Y=1|X_{1},X_{2},X_{3},{\text{PRS}}_{w}\right)\right]=\beta_{0}+\beta_{1}X_{1}+\beta_{2}X_{2}+\beta_{\text{PC}}PC\beta_{g}{\text{PRS}}_{w},$$

where β_*0*_ is the intercept, and β_1_,β_2_,β_PC_=(β_*PC1*_,…,β_*PC10*_), and β_*g*_ are the regression coefficients for *X*_1_,*X*_2_,**PC**,andPRS_*w*_. Then, the AUCs could be calculated with trapezoids (Fawcett, 2006), and their 95% confidence intervals (CI) could be computed by Delong’s method (DeLong et al., 1988). Both AUC and their CI could be implemented directly by the “pROC” package^3^ within R 3.6.3^4^. More details about this package are provided elsewhere (Robin et al., 2011). The best score created in the testing dataset carried forward into subsequent validation step.

### Statistical Analysis in Validation Dataset

Baseline characteristics of the study population were described as means ± standard deviations (M ± SD) or percentages. Two independent sample *t*-test or chi-square test was used to compare the baseline characteristics between the UKB testing and validation datasets. Wilcoxon signed-rank test was applied to give more information about the difference of PRSs between the individuals with T2D and individuals without T2D. The relationship between PRS and T2D was determined in the UKB validation dataset based on logistic regression model adjusting for sex, age, and the first 10 principal components of ancestry (*model*_*1*_), which can be represented as,

T2D∼PRS+sex+age+PC.

We stratified 274,029 participants in the UKB validation dataset as 100 groups according to the percentiles of the PRS, and then, the prevalence of T2D could be determined within each group.

To further observe the contribution of PRS, sex, age, physical measurements, and other clinical risk factors to T2D, we provided other four types of prediction models:

(1) $$m\,o\,d\,e\,l_{2}:\mathrm{T2D}∼\text{sex}+\mathrm{age}+\text{PC};$$

(2) $$m\,o\,d\,e\,l_{3}:\mathrm{T2D}∼\text{PRS};$$

(3) $$model_{\text{4}}:\text{T2D}\sim \text{sex}+\text{age}+PC+\text{BMI}+\text{GL}+\text{CL}+\text{HDL}+\text{LDL}+\text{TL}+\text{WC}+\text{DBP}+\text{SBP};$$

(4) $$model_{\text{5}}:\text{T2D}\sim \text{PRS}+\text{sex}+\text{age}+PC+\text{BMI}+\text{GL}+\text{CL}+\text{HDL}+\text{LDL}+\text{TL}+\text{WC}+\text{DBP}+\text{SBP}.$$

We have checked and did not find the presence of collinearity among the above variables. All of the above statistical analyses were conducted using R version 3.6.3 software.

## Results

A total of 456,451 participants collected in UKB were divided into the UKB testing dataset (*n* = 182,422) and the validation dataset (*n* = 274,029) randomly. The mean ages of participants were 57 years old, and 54% were female in both testing and validation datasets. There were nearly 5.494% (*n* = 10,023) participants who were cases in the testing dataset and 5.575% (*n* = 15,277) in the validation dataset. All of these factors were comparable at baseline. The details of baseline characteristics are shown in Table 1.

**TABLE 1:** Baseline characteristics of the UK Biobank (UKB) testing dataset and the UKB validation dataset (*M* ± *SD* or %).

Variable	UKB testing (*n* = 182,422)	UKB validation (*n* = 274,029)	Statistics and *p-*value
**Sex**			
Male (%)	83,200 (45.609)	125,670 (45.860)	*x*^2^ = 2.783, *p* = 0.095
Female (%)	99,222 (54.391)	148,359 (54.140)	
Age (years)	56.777 ± 8.020	56.809 ± 8.009	*t* = −1.341, *p* = 0.179
**Physical measurements**			
BMI (kg/m^2^)	27.388 ± 4.758	27.404 ± 4.765	*t* = −1.087, *p* = 0.277
WC (cm)	90.250 ± 13.485	90.306 ± 13.505	*t* = −1.135, *p* = 0.175
DBP (mmHg)	82.174 ± 10.311	82.171 ± 10.313	*t* = −0.118, *p* = 0.906
SBP (mmHg)	139.924 ± 19.000	139.917 ± 19.000	*t* = −0.116, *p* = 0.908
**Clinical factors**			
CL (mmol/L)	5.711 ± 1.115	5.710 ± 1.117	*t* = −0.314, *p* = 0.753
GL (mmol/L)	5.119 ± 1.134	5.118 ± 1.132	*t* = 0.150, *p* = 0.881
TL (mmol/L)	1.753 ± 1.002	1.753 ± 1.000	*t* = −0.010, *p* = 0.992
HDL (mmol/L)	1.452 ± 0.357	1.453 ± 0.358	*t* = −0.625, *p* = 0.532
LDL (mmol/L)	3.556 ± 0.839	3.556 ± 0.841	*t* = −0.083, *p* = 0.934
**Type 2 diabetes**			
Case (%)	10,023 (5.494)	15,277 (5.575)	*x*^2^ = 1.342, *p* = 0.247
Control (%)	172,399 (94.506)	258,752 (94.425)	

*BMI, body mass index; CL, cholesterol level; DBP, diastolic blood pressure; GL, glucose level; HDL, high-density lipoprotein; LDL, low-density lipoprotein; SBP, systolic blood pressure; TL, triglyceride level; WC, waist circumference.*

To obtain an optimal PRS model, we generated a total of 16 candidate PRS models implemented by PRSice-2 software. We evaluated the performance of these 16 PRS models in the UKB testing dataset and chose the best one for further validation analysis. The AUCs of these 16 candidate PRS models ranged from 0.691 to 0.792 (Table 2). We selected the best PRS model with the highest AUC [AUC = 0.792, 95% CI: (0.787, 0.796)] based on 25,454 SNPs when *p*≤ 5× 10^−2^ and *r*^2^ < 0.2. The AUCs of different ratios of the testing and validation datasets are shown in Table 3. We can see that the AUCs of different ratios were very close to each other, which ranged from 0.791 to 0.795. The AUC of the 40–60% ratio had the best performance in the validation dataset [AUC = 0.795, 95% CI: (0.790, 0.800)]. Additional details of PRS model construction, testing, and validation are provided in Figure 1.

**TABLE 2:** The predictive power of candidate polygenic risk score (PRS) models for type 2 diabetes (T2D).

Tuning parameter	SNP number	AUC (95% CI)
*p*≤ 5× 10^−8^ and *r*^2^ < 0.2	363	0.706 (0.701–0.711)
*p*≤ 5× 10^−8^ and *r*^2^ < 0.4	486	0.702 (0.697–0.707)
*p*≤ 5× 10^−8^ and *r*^2^ < 0.6	670	0.696 (0.691–0.701)
*p*≤ 5× 10^−8^ and *r*^2^ < 0.8	957	0.691 (0.686–0.697)
*p*≤ 5× 10^−6^ and *r*^2^ < 0.2	750	0.715 (0.710–0.720)
*p*≤ 5× 10^−6^ and *r*^2^ < 0.4	1,013	0.709 (0.704–0.714)
*p*≤ 5× 10^−6^ and *r*^2^ < 0.6	1,335	0.701 (0.696–0.706)
*p*≤ 5× 10^−6^ and *r*^2^ < 0.8	1,853	0.696 (0.691–0.701)
*p*≤ 5× 10^−4^ and *r*^2^ < 0.2	2,616	0.736 (0.732–0.741)
*p*≤ 5× 10^−4^ and *r*^2^ < 0.4	3,394	0.726 (0.721–0.731)
*p*≤ 5× 10^−4^ and *r*^2^ < 0.6	4,299	0.715 (0.710–0.720)
*p*≤ 5× 10^−4^ and *r*^2^ < 0.8	5,690	0.708 (0.703–0.713)
*p*≤ 5× 10^−2^ and *r*^2^ < 0.2	**25,454**	**0.792 (0.787–0.796)**
*p*≤ 5× 10^−2^ and *r*^2^ < 0.4	32,600	0.782 (0.777–0.787)
*p*≤ 5× 10^−2^ and *r*^2^ < 0.6	40,001	0.771 (0.766–0.776)
*p*≤ 5× 10^−2^ and *r*^2^ < 0.8	50,224	0.760 (0.755–0.765)

*AUC was determined using a logistic regression model adjusted for sex, age, and the first 10 principal components of ancestry. The highest AUC is denoted by the bold values.*

**TABLE 3:** Area under the receiver operating characteristics curves (AUCs) of different ratios of the testing and validation dataset when *p*≤ 5× 10^−2^ and *r*^2^ < 0.2.

Dataset	30–70%	40–60%	50–50%	60–40%	70–30%
Testing	0.791	0.792	0.794	0.795	0.794
	(0.781–0.791)	(0.787–0.796)	(0.790–0.800)	(0.791–0.799)	(0.790–0.799)
Validation	0.794	0.795	0.793	0.792	0.791
	(0.790–0.799)	(0.790–0.800)	(0.789–0.797)	(0.787–0.796)	(0.781–0.791)

*AUC was determined using a logistic regression model adjusted for sex, age, and first 10 principal components of ancestry.*

**FIGURE 2:**
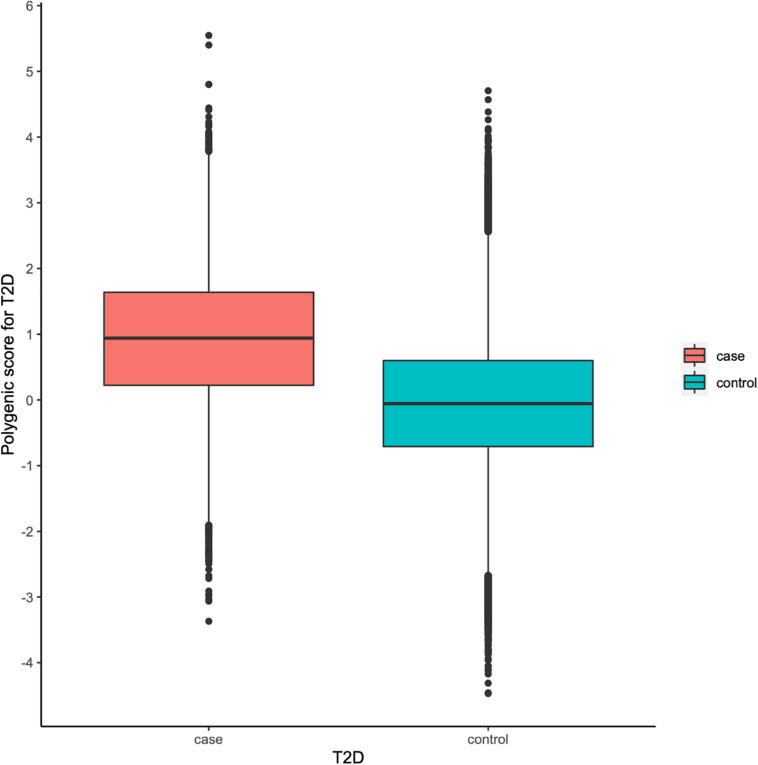
Polygenic risk score (PRS) among type 2 diabetes (T2D) cases versus controls in the UK Biobank (UKB) validation dataset.

**FIGURE 3:**
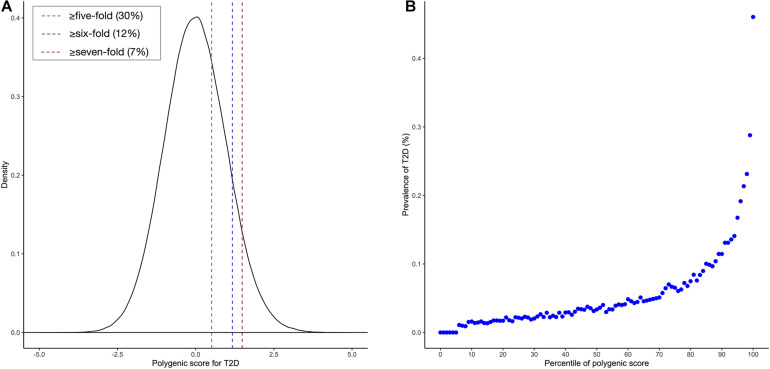
Risk for type 2 diabetes (T2D) according to polygenic risk score (PRS). **(A)** Distribution of PRS for T2D in the UK Biobank (UKB) validation dataset (*n* = 301,736). The *x*-axis represents PRS for T2D, which was scaled to have zero mean and one standard deviation. Dotted lines reflect the proportion of the population with five-, six-, and seven-fold increased risk versus the remainder of the population, respectively. The odds ratio was assessed in a logistic regression model adjusting for sex, age, and the first 10 principal components of ancestry. **(B)** Prevalence of T2D according to 100 groups of the UKB validation dataset stratified according to the percentile of the PRS for T2D.

To facilitate interpretation, we scaled PRS to have zero mean and one standard deviation. We investigated whether our PRS model could identify individuals at high T2D risk. Figure 2 showed that the median of the standardized PRS was 0.941 for individuals with T2D versus −0.056 for individuals without T2D, a difference of 0.997 (*p* < 0.00001). From Figure 3A, we found that the standardized PRS approximated a normal distribution across the population with the empirical risk of T2D rising sharply in the right tail of the distribution. The PRS model identified nearly 30% of the population at greater than or equal to fivefold risk, 12% of the population at greater than or equal to sixfold risk, and the top 7% of the population at greater than or equal to sevenfold increased risk for T2D shown in Figure 3A. Then, we stratified the population according to the percentiles of the PRS and defined the top 10 percentiles as “high risk” group while the bottom 10 percentiles as “low risk” group. Figure 3B showed the prevalence of T2D increases with the percentiles of the PRS model. There were 5,642 (18.698%) cases in “high risk” group among 30,174 individuals, while only 282 (0.935%) cases in the “low risk” group, corresponding to a nearly 20-fold increase in the risk of T2D comparing the top 10 percentiles versus the bottom 10 percentiles.

We further investigated the contribution of polygenic predictor, sex, age, physical measurements, and clinical factors in identifying individuals at high risk of T2D. Table 4 showed that the AUCs of *model*_*3*_, which only included PRS into the prediction model without adjusting for any other covariates, was 0.749 [95% CI: (0.744,0.754)] in the testing dataset and 0.755 [95% CI: (0.752, 0.755)] in the validation dataset. Interestingly, if only considering sex, age, and the first 10 principal components of ancestry into the model, the AUC was 0.667 [95% CI: (0.663, 0.672)]. After adding PRS, the AUC reached 0.795 [95% CI: (0.790, 0.800)], which increased about 13% than *model*_*2*_. The AUC of *model*_*4*_ (i.e., considering sex, age, ***PC***, BMI, WC, DBP, SBP, GL, CL, HDL, LDL, and TL simultaneously) was 0.880 [95% CI: (0.878, 0.888)] and raised to 0.901 [95% CI: (0.897, 0.904)] in the validation dataset when adding PRS into the model. In brief, the polygenic score indeed helps to identify high-risk individuals for T2D, while the role of T2D-related covariates could also help increase prediction accuracy. As showed in Table 5, PRS, sex, age, physical measurements, and most clinical factors were all significantly associated with T2D (*p* < 0.0001).

**TABLE 4:** Area under the receiver operating characteristics curve (AUC) of different models in the testing and validation dataset.

Dataset	Mean	*model*_*2*_	*model*_*3*_	*model*_*1*_	*model*_*4*_	*model*_*5*_
Testing	−0.003	0.671 (0.666–0.676)	0.749 (0.744–0.754)	0.792 (0.787–0.796)	0.886 (0.882–0.889)	0.902 (0.899–0.905)
Validation	−0.003	0.667 (0.663–0.672)	0.755 (0.752–0.755)	0.795 (0.790–0.800)	0.882 (0.878–0.888)	0.901 (0.897–0.904)

**model*_*1*_: AUC was determined using a logistic regression model adjusted for sex, age, and the first 10 principal components of ancestry. *model*_*2*_: AUC was determined using a logistic regression model only considering sex and age. model_3_ : AUC was determined using a logistic regression model only considering genome-wide polygenic score. *model*_*4*_: AUC was determined using a logistic regression model considering demographic factors, physical measurements, and clinical factors. model_5_ : AUC was determined using a logistic regression model adjusted for sex, age, body mass index, waist circumference, diastolic blood pressure, systolic blood pressure, glucose level, cholesterol level, high-density lipoprotein, low-density lipoprotein, triglyceride level, and the first 10 principal components of ancestry.*

**TABLE 5:** Parameter estimations under *model*_*5*_ in validation dataset.

Variables	Estimate beta	Stand error	*Z*	*p*-value
(Intercept)	24.500	0.495	49.474	< 2× 10^−16^
PRS	12370.000	167.400	73.943	< 2× 10^−16^
CL	−0.591	0.057	−10.377	< 2× 10^−16^
HDL	0.051	0.063	0.876	0.381
LDL	0.010	0.068	0.140	0.888
TL	0.285	0.013	21.826	< 2× 10^−16^
Sex	−0.214	0.028	−7.731	1.070× 10^−14^
WC	0.045	0.002	28.356	< 2× 10^−16^
BMI	0.036	0.004	9.325	< 2× 10^−16^
Age	0.060	0.002	38.401	< 2× 10^−16^
DBP	−0.018	0.001	−13.928	< 2× 10^−16^
SBP	0.005	0.001	7.626	2.410× 10^−16^
GL	0.449	0.006	69.917	< 2× 10^−16^
PC10	0.020	0.004	4.726	2.280× 10^−16^

*BMI, body mass index; CL, cholesterol level; DBP, diastolic blood pressure; GL, glucose level; PRS, genome-wide polygenic score; HDL, high-density lipoprotein; LDL, low-density lipoprotein; SBP, systolic blood pressure; TL, triglyceride level; WC, waist circumference.*

## Discussion

Our results showed that the AUC of the best PRS model was 0.795 after adjusting for sex, age, and the first 10 principal components of ancestry. It demonstrated that the PRS was really helpful for identifying individuals at high risk of developing T2D. Meanwhile, the distributions of the PRS in cases and controls were substantially different from each other, i.e., the median PRS of cases (0.941) was much higher than that of the controls (−0.056). Moreover, about 30% of participants were at greater than or equal to fivefold increased risk of developing T2D, 12% were at greater than or equal to sixfold risk, and the top 7% were at greater than or equal to sevenfold increased risk. Particularly, the stratified PRS according to their percentiles showed that the “high-risk” group is strongly associated with the risk of T2D.

The above results suggest that our PRS model can be used as a powerful tool in identifying individuals at high risk of T2D; improved previous studies that summarized in Table 6. The AUC of the PRS model assessed with only three SNPs that had been published to predispose to T2D in 6,078 individuals was 0.571 (Weedon et al., 2006). After including more SNPs, Lango et al. (2008) constructed a PRS model with 18 SNPs and obtained an AUC of 0.600 (Lango et al., 2008). A later study with 22 SNPs had an AUC of 0.570 (Chatterjee et al., 2013) and allowed for the identification of 3.0% of the population at twofold or higher than average risk for T2D. Notably, the above three studies with smaller sample sizes (range from 4,907 to 39,117), and a smaller number of SNPs (range from 3 to 22) had relatively poor predictive performance compared to our study (AUC = 0.755) with 25,454 SNPs among 274,029 individuals.

**TABLE 6:** A comprehensive comparison with other researches.

Year	SNP	N	Case\Control	Case/N (%)	Dataset	AUC	Ethnicity	Covariates
Weedon et al., 2006	3	6,078	2,409\3,669	39	UKCS	0.571	British	–
Lango et al., 2008	18	4,907	2,309\2598	47	GoDARTS	0.600	Scotland	–
Lango et al., 2008	18	4,907	2,309\2598	47	GoDARTS	0.800	Scotland	Age, BMI, and sex
Lyssenko et al., 2008	16	18,831	2,201\16,630	11.68	MPP and BS	0.750	Finland	Sex, age, family history, BMI, BP, TL, and GL
Chatterjee et al., 2013	22	39,117	130\38,987	0.3	–	0.570	Caucasian	–
Chatterjee et al., 2013	22	39,117	130\38,987	0.3	–	0.740	Caucasian	Sex, age, and family history
Läll et al., 2017	1,000	10,273	1,181\9,092	11.5	EBC	0.74	Estonia	Sex and age
Läll et al., 2017	1,000	10,273	1,181\9,092	11.5	EBC	0.767	Estonia	Sex, age, and BMI
Läll et al., 2017	1,000	10,273	1,181\9,092	11.5	EBC	0.790	Estonia	Sex, age, BMI, BP, GL, physical activity, smoking, and food consumption
Khera et al., 2018	6,917,436	288,978	5,853\283,125	2	UKB	0.730	British	Sex and age
–	25,454	274,029	18,176\283,560	6	UKB	0.755	British	–
–	25,454	274,029	18,176\283,560	6	UKB	0.795	British	Sex and age
–	25,454	274,029	18,176\283,560	6	UKB	0.901	British	Sex, age, WC, BMI, SBP, DBP, GL, CL, TL, HDL, and LDL

In addition, we highlight the role of non-genetic risk factors, i.e., sex, age, physical measurements, and clinical factors. When adjusting for sex and age, Meigs et al. (2008) obtained an AUC of 0.581 among 2,776 individuals, Vassy et al. (2014) provided an AUC of 0.726 among 11,883 people, and the AUC of Läll et al. (2017) reached 0.740. Interestingly, the study that handled nearly 7 million variants in 288,978 individuals only generated an AUC of 0.730 after adding sex and age, which was smaller than ours (0.795) including only 25,454 SNPs (Khera et al., 2018). They further reported that 3.5% of the population had inherited a genetic predisposition that conferred greater than or equal to threefold increased risk for T2D, 0.2% of the population greater than or equal to fourfold, and 0.05 of the population greater than or equal to fivefold. Their study differs from ours in four aspects. First, our study has larger sample size (456,451 versus 409,258). Second, we first perform SNP selection based on genome-wide association *p*-values (*p*≤ 5× 10^−2^) so that we included more predictive SNPs (25,454) and avoided spurious SNPs into our PRS model. Third, they used the first 4 principal components of ancestry, while we used the first 10 principal components of ancestry for a better control of population stratification. Fourth, we generated PRS based on the more computationally efficient and scalable PRSice-2 software, while they used LDpred program (Ripke et al., 2015), which is much slower than PRSice-2. Those differences explain why our PRS model has better predictive power. Certainly, we also tried to incorporate more non-genetic risk factors, and the AUC increased from 0.755 to 0.901. Our study is thus more accurate in identifying individuals at low and high risk of developing T2D.

Our study has multiple strengths. First, we construct the PRS model based on the UKB dataset, which is one of the largest prospective cohort studies with comprehensive and abundant personal information, as well as high-quality genotyping data in the world. Second, we choose SNPs into our PRS model based on our proposed three-step filtering procedure. This approach is simple to implement and has a very good prediction performance. Third, we include new physical measurements and clinical factors (i.e., WC, DBP, HDL, and LDL) in our predictive model to increase prediction accuracy. Fourth, we adopted a new PRS software PRSice-2, which has been shown to outperform other competing methods and software in terms of prediction accuracy and computational speeds (Choi and O’Reilly, 2019).

Although the present study has made important contributions in identifying individuals with increased risk of developing T2D; however, there exists one major limitation. Individuals in the UKB dataset are primarily European ancestry; the specific PRS calculated here may not have optimal predictive power for other ethnic groups because the allele frequencies, LD patterns, and effect sizes of common SNPs may be different across populations with different ethnic backgrounds.

In conclusion, our findings show that the PRS model is highly predictive of T2D risk even based on genetic data only, and the prediction accuracy improves after including non-genetic risk factors, suggesting that our PRS model can be used as a powerful tool for preventive T2D screening.

## Data Availability Statement

The original contributions presented in the study are included in the article/supplementary material, further inquiries can be directed to the corresponding authors.

## Author Contributions

ZL and TH initiated the study. WL developed the strategy, performed the data analysis, and completed the manuscript writing. ZZ and WW contributed to the data collection and manuscript reservation. All authors contributed to the article and approved the submitted version.

## Conflict of Interest

The authors declare that the research was conducted in the absence of any commercial or financial relationships that could be construed as a potential conflict of interest.

## Footnotes

1 http://biobank.ctsu.ox.ac.uk/crystal/

2 http://diagram-consortium.org/

3 https://cran.r-project.org/web/packages/pROC/index.html

4 https://cran.r-project.org/bin/macosx/
